# Supplementary material for: Early-life stress impairs postnatal oligodendrogenesis and adult emotional behaviour through activity-dependent mechanisms
Source: Mol Psychiatry. 2019 Aug 22;25(6):1159–74. doi: 10.1038/s41380-019-0493-2 (PMC7244403; doi:10.1038/s41380-019-0493-2)
Supplement: Supplementary file 1 — Legends to supplementary material [file 41380_2019_493_MOESM1_ESM.docx]

**Supplementary Figure 1: RNA sequencing of P15 mPFC after Maternal Separation reveals an increase un myelin-related transcripts and a decrease in the expression of immediate early genes.** (A,B) GO Biological Process of genes upregulated (B) or downregulated (C) in P15 mPFC of MS versus SFR animals (q<0.05, change >30%). (C) Image of Western Blot gel of mPFC extracts from P15 SFR and MS brains stained with anti-Plp1 (red) and anti-Tuj1 (green) antibodies. (B,C) Graphs show the ratio of Plp1 band intensity relative to Tuj1 band intensity in P15 (n=5 SFR/5 MS) and adult (n=4 SFR/4 MS) mPFC , confirming a transient increase of this myelin protein specific to P15 MS mPFC. **p<0.01

**Supplementary Figure 2: P9 mPFC of MS animals show a decrease in cFos labelling before the oligodendrogenesis changes.** (A) Scheme illustrates the time line of the experiments : SFR animals never exposed to separation were compared to MS animals exposed to daily to maternal separation from P2 to P8 and killed 24h after the last separation. (B) Histogram shows cFos cells/mm2 in P9 mPFC of MS (n=3) and SFR (n=4) animals. (C) Graph shows the density of OPCs (PDGFRα+ Olig2+, dark orange) and OLs (PDGFRα+ Olig2+, light orange) in P9 mPFC of SFR (n=5) and MS (n=5) animals. **p<0.01

**Supplementary Figure 3: DREADD-dependent changes in neuronal activity in the developing mPFC.** (A, B) Patch Clamp recordings of thick-tufted layer 5 pyramidal neurons were done in the prelimbic cortex of P9-P11 pups injected with AAV8-hSyn-hM3D(Gq)-mCherry virus. This revealed an increase in neuronal excitability upon exposure to CNO (30µM, n=13 cells from 5 mice) or clozapine (100nM, n= 8 cells from 4 mice) as compared to controls (n=13 cells from 4 mice). (C,D) Patch Clamp recordings of the same neuronal cell-type in pups injected with AAV8-hSyn-hM4D(Gi)-mCherry. This showed no difference in

neuronal excitability upon exposure to CNO (n=10 cells from 2 mice) or clozapine (n=13 cells from 3 mice) as compared to control (n=13 cells from 3 mice). (E) Schematic depicting the experimental design for measuring the outward current in voltage-clamped hM4Di-expressing neurons. hM4Di-mCherry+ thick-tufted pyramidal neurons in PFC layer 5 were voltage-clamped at a holding potential of -65mV. (F) Example trace showing an outward current elicited by the application of CNO (30 µM) and inhibited by Ba2+ (100µM), a blocker of GIRK channels. (G) Summary scatter plot of CNO-induced currents (Mann-Whitney test, n=11 cells from 8 mice). (H) Schematic depicting the experimental design for investigating putative changes in the probability of glutamate release from hM4Di-expressing neurons. Viral vectors encoding hM4Di-mCherry and ChR2-YFP were co-injected in deep layers of the PFC. ChR2-YFP^-^ thick-tufted pyramidal neurons located in the vicinity of hM4Di-mCherry^+^ YFP^+^ neurons in PFC layer 5 were voltage-clamped at a holding potential of -65mV. ChR2 was activated with a blue light-emitting LED. (I) Example traces showing EPSCs evoked by two consecutive light pulses (blue arrowheads) in a neuron recorded in the absence of CNO (top trace), and in another neuron recorded in the presence of CNO 30 mM (bottom trace). (J) Summary histogram of the paired-pulse ratio. Open circles refer to individual recordings (control : n=10 from 3 mice and CNO: n=10 from 3 mice). n.s. not significant, T-Test.

**Supplementary Figure 4:** **Immediate early gene labeling in the mPFC after CNO or SAL injection in vivo.** (A) P8 and P14 mice were perfused 2h after a single ip injection of CNO (5mg/kg) or SAL and mPFC sections were double-immunostained for cell counts. Histograms show the proportions of transfected mCherry+ neurons in the mPFC that are also Egr1+ at P8 (n=5SAL/4CNO) and P14 (n=3SAL/6CNO). (B,C) Brain sections of P15 mice were analyzed 24hrs after daily injections of CNO or SAL from P2 to P14. Histograms show the number of cFos+ neurons according to their expression of the viral mCherry in the mPFC of P15 SFR+hM4Di (B, n=3SAL/4CNO) and MS+hM3Dq (C, n=3SAL/4CNO) animals. *p<0.05, **p<0.01, ***p<0.005.

**Supplementary Figure 5: Early alterations of mPFC activity impairs postnatal growth.** (A, B, C,D) Graphs show the weight of adult males exposed to maternal separation (A, n=14SFR/20MS), SFR+hM4Di (B, n=12SAL/14CNO) , MS+hM3Dq (C, n=18SAL/17CNO) or GFP (D, n=17PNSAL/20PNCNO) paradigms. (E, F,G,H) Graphs show the postnatal weight growth of pups exposed to maternal separation (E, n=22SFR/27MS), SFR+hM4Di (F, n=17SAL/22CNO) MS+hM3Dq (G, n=22SAL/25CNO) or GFP (H, n=18PNSAL/21PNCNO) paradigms. *p<0.05, ***p<0.005.

**Supplementary Figure 6: Early Transient exposure to CNO does not alter anxiety and depression-like behaviors in adult males.** (A) P1 pups were bilaterally injected with AAV-hSyn-GFP in the mPFC and received daily i.p. injections with CNO (5mg/kg) or SAL from P2 to 14 (PNCNP/PNSAL) before undergoing behavioral assessment in adults. We observed no significant difference between PNSAL and PNCNO treated animals in any of the behavioral tests performed in this cohort: (B) distance travelled in the OF (F(1,27)=1.390 p=0.2486, n=15PNSAL/14PNCNO), (C) time spent in the center of the OF (F(1,27)=0.108 p=0.7450, n=14PNSAL/14PNCNO), (D) time spent in the open arm of the EPM (F(1,28)=0.474 p=0.4970, n=13PNSAL/17PNCNO), (E) the number of marbles buried (F(1,287)=0.51 p=0.8234, n=13PNSAL/19PNCNO), (F) floating behavior (F(1,25)=0.589 p=0.45), (G) short term memory (F(1,26)=1,64E-4 p=0.9899), and (H) time spent grooming in the 5min following a sucrose splash (F(1,29)=0.109 p=0.7436, n=12/19).
