# Supplementary material for: Early-life stress impairs postnatal oligodendrogenesis and adult emotional behaviour through activity-dependent mechanisms
Source: Mol Psychiatry. 2019 Aug 22;25(6):1159–74. doi: 10.1038/s41380-019-0493-2 (PMC7244403; doi:10.1038/s41380-019-0493-2)
Supplement: Supplementary file 2 — supplementary figures [file 41380_2019_493_MOESM2_ESM.pdf]

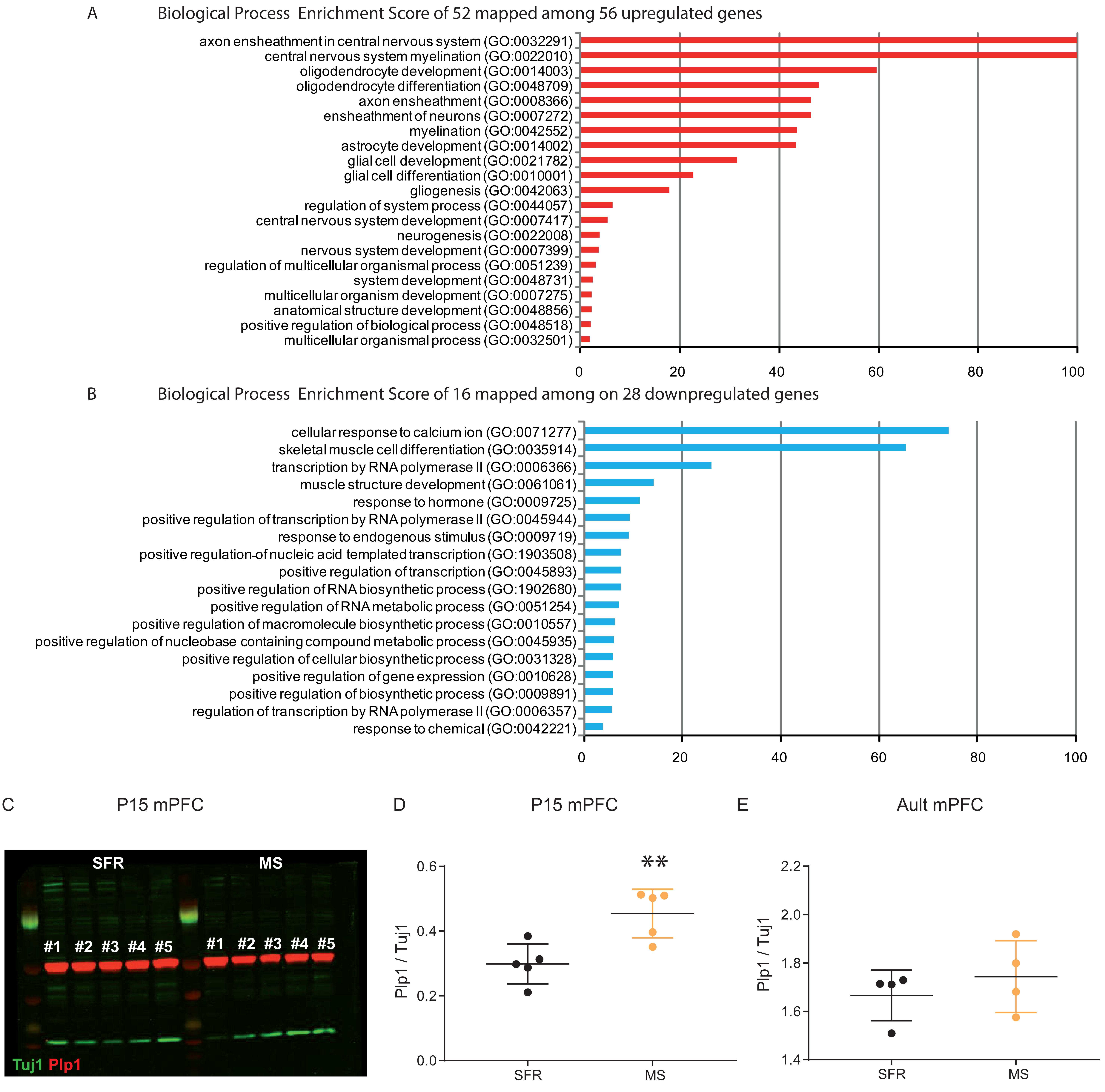

Supplementary Figure 1

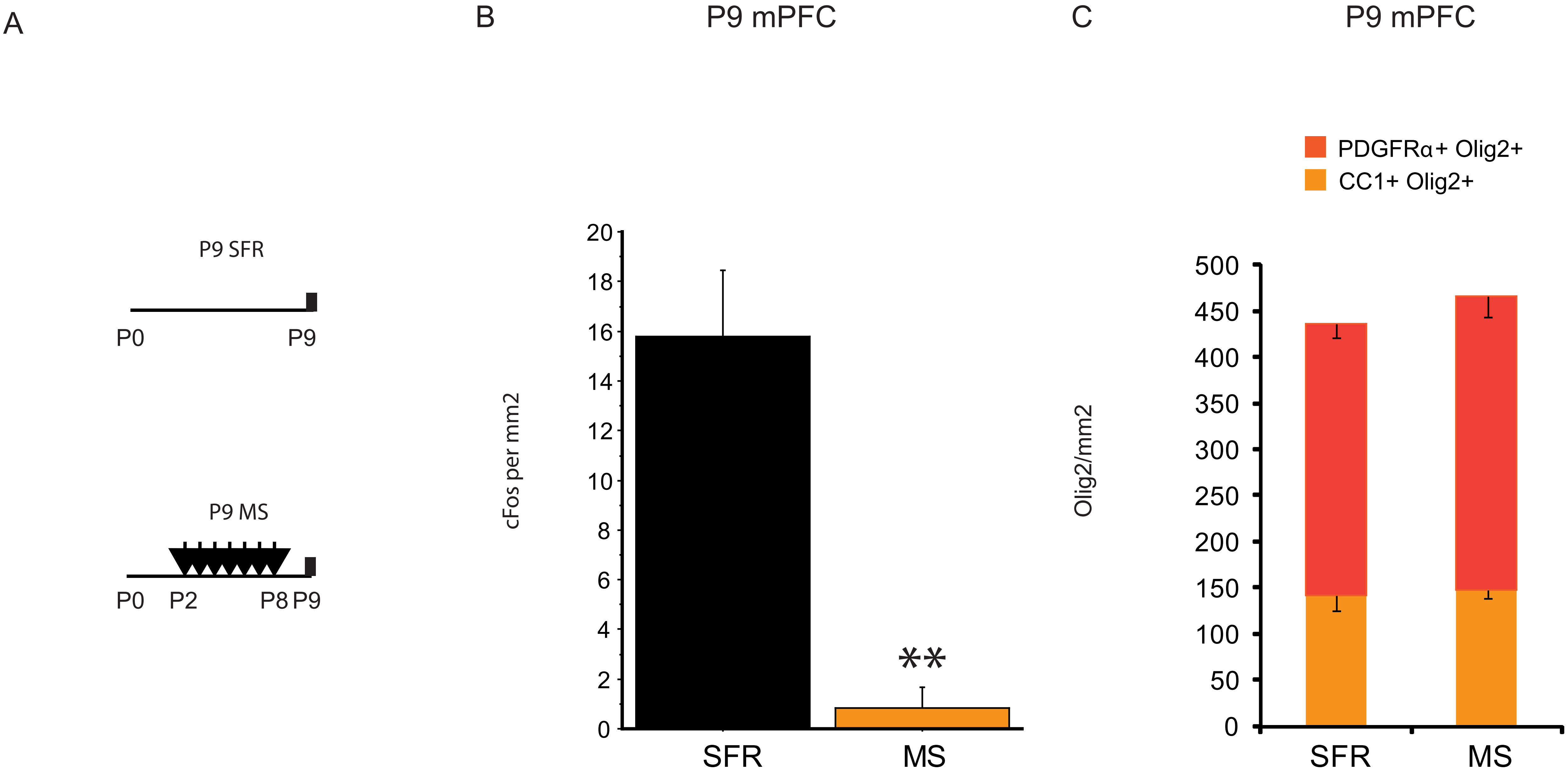

. **Supplementary Figure 2**

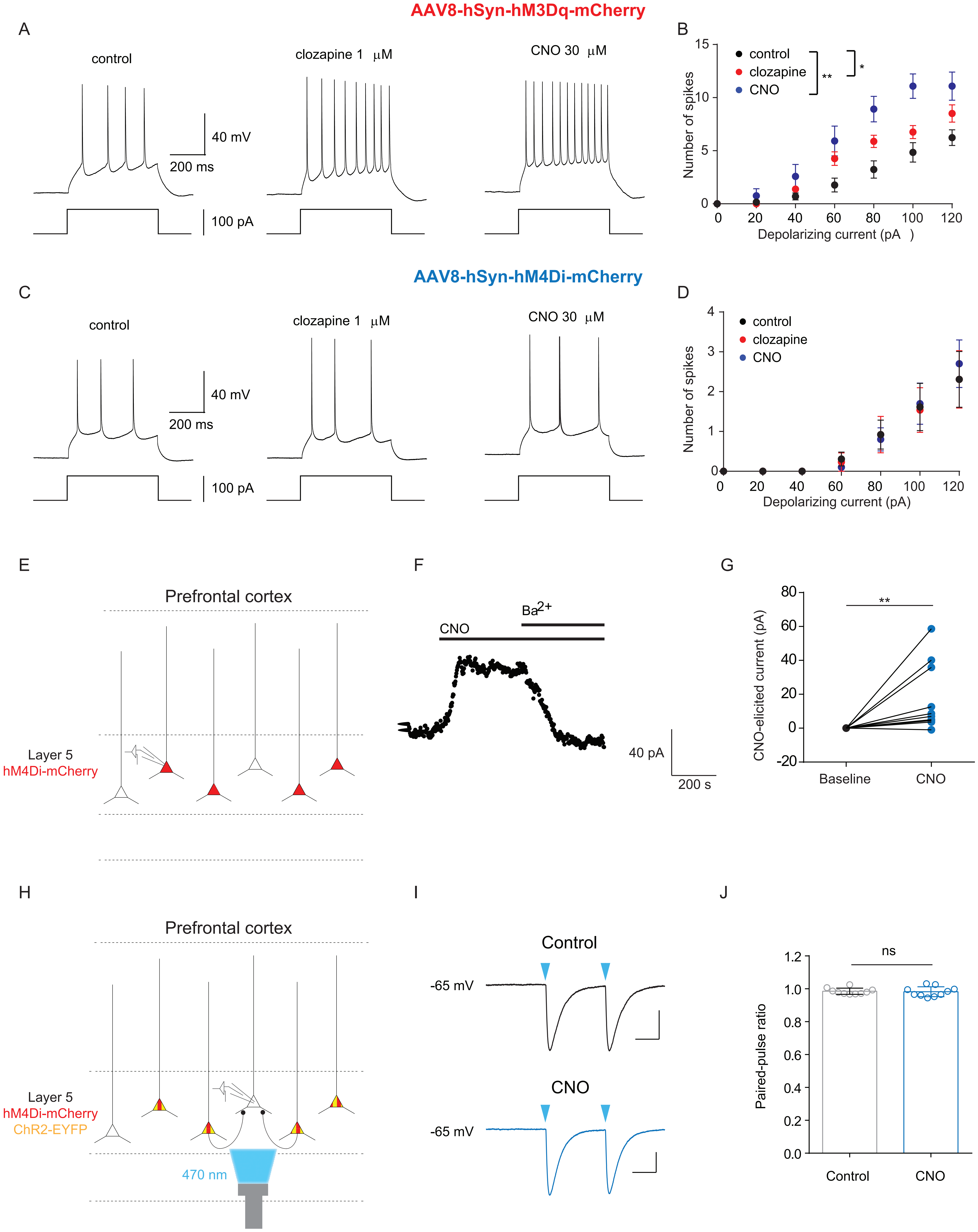

Supplementary Figure 3

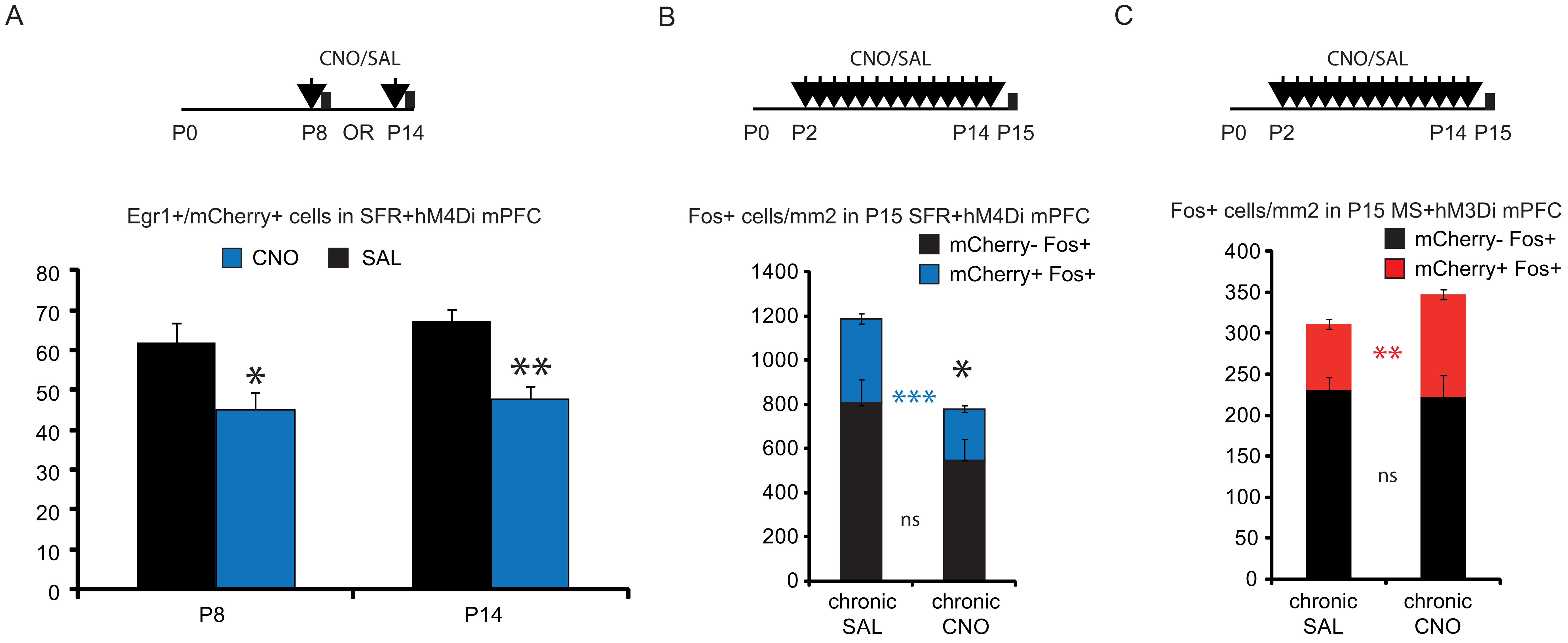

Supplementary Figure 4

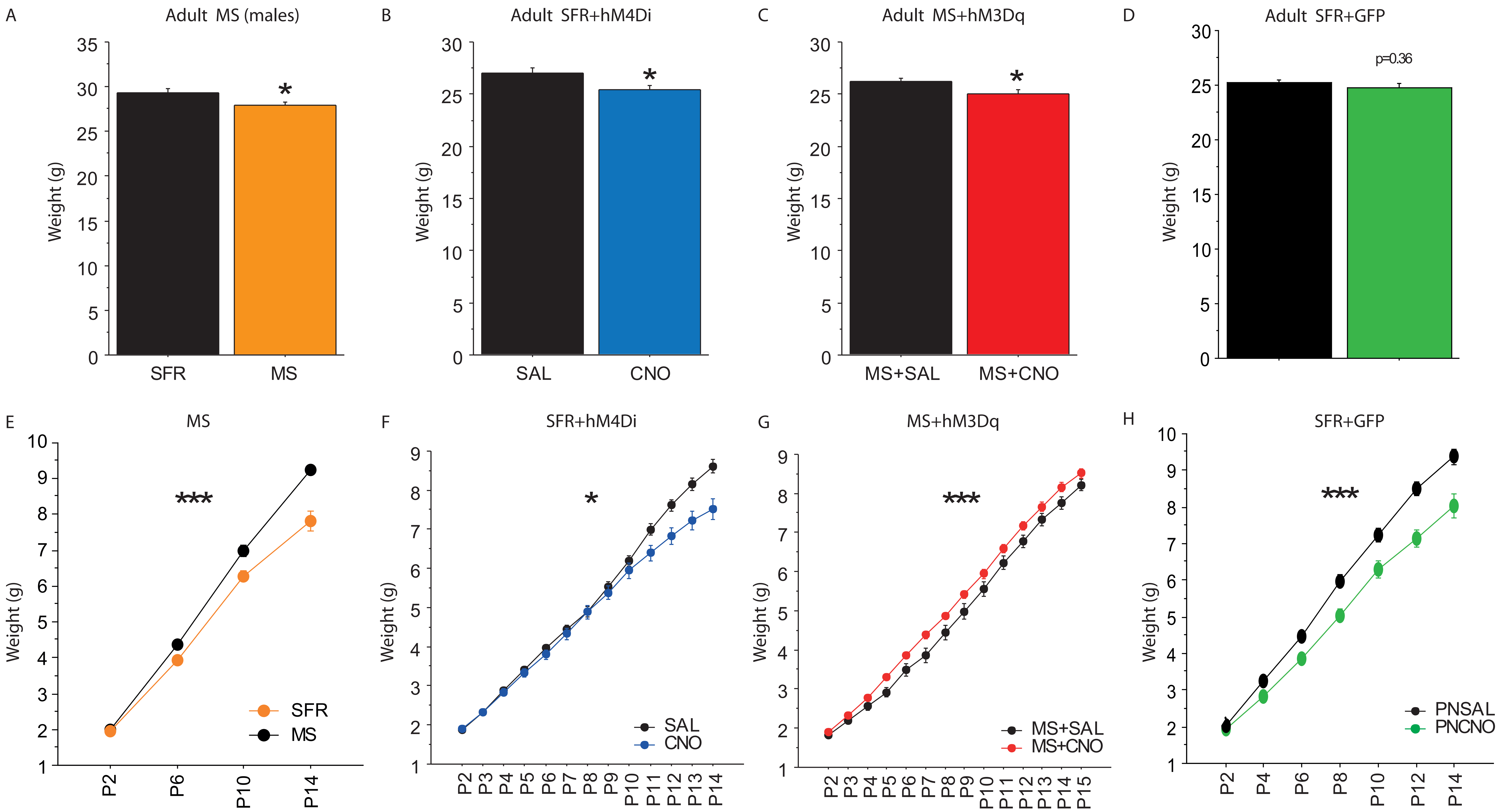

Supplementary Figure 5

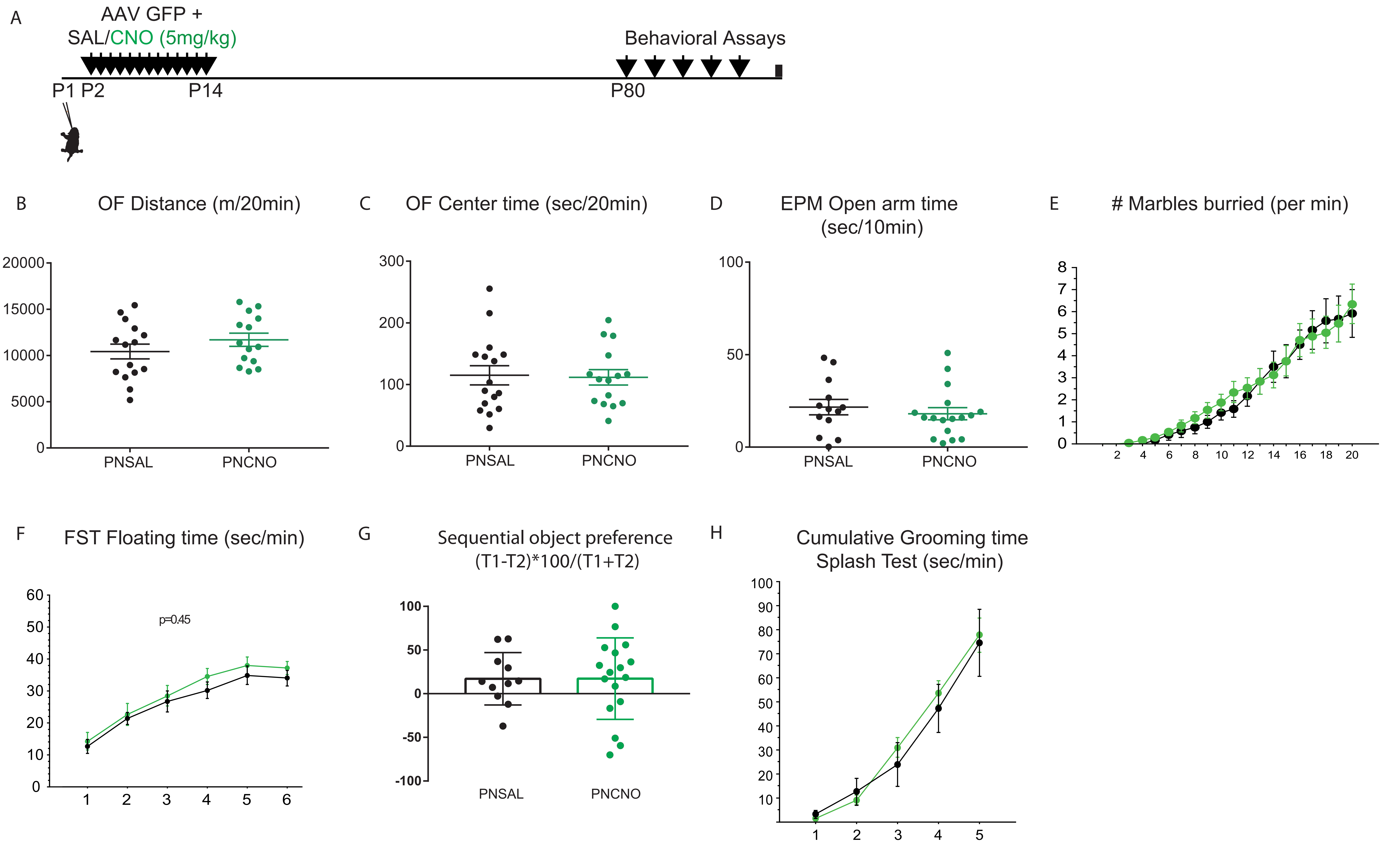

Supplementary Figure 6

Supplementary Table 1: Differentially Expressed Genes after MS in P15 mPFC (q<0.05)

Associated Gene Name	LOG2 reads SFR	LOG2 FC MS	p value	qvalue
Egr2	9,02375383	-1,126006315	6,5E-21	1,08E-16
Nr4a1	11,41156395	-0,730383731	2,78E-17	2,3E-13
Tspan2	9,762062828	0,720010208	5,77E-12	2,39E-08
Ugt8a	9,517559001	0,797271877	1,1E-11	3,64E-08
Scn4b	6,648903197	0,918592049	1,57E-11	4,35E-08
Egr4	10,30941143	-0,528635473	1,11E-10	0,000000264
Junb	10,33996104	-0,615224159	2,17E-10	0,00000045
Gjc3	7,652885845	0,632713474	2,52E-10	0,000000464
Cd4	1,460527981	2,42098154	3,26E-10	0,000000541
Fos	9,558588375	-0,866091025	7,89E-09	0,00000872
Ier2	8,048671807	-0,529595203	8,93E-09	0,00000925
Gm28437	5,221248586	2,967628059	9,59E-09	0,00000936
Arc	11,95602984	-0,758643317	0,000000011	0,0000101
St18	4,613254194	1,195836658	1,21E-08	0,0000106
Lpar1	7,933875798	0,678589785	2,45E-08	0,0000203
Prlr	4,532633011	-2,001629386	0,000000213	0,000139417
Plp1	12,60011609	0,83878217	0,000000219	0,000139417
Ermn	6,62903443	0,900631705	0,000000396	0,000234541
Nts	4,842267279	-0,885197351	0,000000442	0,000252899
Myrf	8,681915138	0,551147447	0,000000568	0,00030364
Mag	10,05480175	0,840152772	0,000000562	0,00030364
Fosb	8,49062432	-0,685752069	0,000000633	0,000327936
Eomes	2,087515124	1,912468164	0,000000867	0,000435788
Gm13066	5,526470294	-0,620020444	0,00000102	0,000487098
Mobp	11,11124947	0,923463751	0,00000103	0,000487098
Adamts4	7,72845064	0,620668354	0,00000139	0,000604872
Plxnb3	8,43739156	0,56462054	0,00000235	0,000950779
Nr1i3	6,054142033	-0,500907386	0,00000305	0,001204231
Clic6	3,997992816	-1,042206393	0,00000355	0,00133789
Mog	7,405370509	0,755453762	0,00000348	0,00133789
Ppp1r1b	9,604652043	0,646620602	0,00000391	0,00144239
Fa2h	7,863464422	0,729353204	0,00000519	0,001869891
Cldn11	9,659859378	0,804930069	0,00000541	0,001910377
Pde10a	10,19689363	0,396871906	0,00000609	0,002055066
Ntf3	4,744423666	-0,769804677	0,0000074	0,002359843
Cnksr3	7,061357185	0,581960942	0,00000789	0,002467867
Gsn	9,496893965	0,657798115	0,00000835	0,002564693
Erbb3	6,63106478	0,638710738	0,00000892	0,002639535
Thbs4	4,459510775	0,949831152	0,00001	0,002813664
1700016P03Rik	6,371516477	-0,596772857	0,0000112	0,003089316
Nkx6-2	4,930460027	0,915398433	0,0000142	0,003810841
1810049J17Rik	5,285190416	-0,858678538	0,0000165	0,004270157
Ddc	5,586004781	0,734670344	0,0000173	0,004350241
Cnp	11,27643462	0,618838219	0,0000188	0,004645962
Gm4409	5,519508287	-0,561333921	0,0000192	0,004693585
Hipk4	5,24654989	-0,544687464	0,0000233	0,005590229
Rnf122	7,312323235	0,544814982	0,0000241	0,005713819
Fbxo32	6,923939992	0,560622746	0,0000248	0,005781423
Rasgef1b	9,64835912	0,410577466	0,000028	0,006371634
Mbp	13,91411933	0,777938255	0,0000299	0,006711352
Adora2a	6,022648217	0,584921755	0,0000367	0,008088682
Elovl7	6,926672412	0,620958585	0,0000371	0,008088682
Slc45a3	3,781312709	1,000140868	0,0000411	0,008743406
1700047M11Rik	5,468802989	0,833857795	0,0000434	0,009119293
Gm15337	4,768984581	-0,682221596	0,0000452	0,009363815
Tpx2	5,948555967	0,609754926	0,0000526	0,010621885
Mal	8,407818278	0,903169585	0,0000519	0,010621885
4930404N11Rik	5,227762848	-0,734521179	0,0000583	0,011512271
Erbb2ip	9,421443092	0,409296601	0,0000672	0,012804779
Anln	5,864631814	0,674209603	0,0000999	0,018411831
Plekhh1	7,251100575	0,491337231	0,0000997	0,018411831
Mob3b	6,265102048	0,640626217	0,000105023	0,018932366
Tmem88b	8,052224336	0,695812026	0,000105028	0,018932366

**Sgk2**	3,210154152	1,328355184	0,000117092	0,020880206
**Carhsp1**	7,645317422	0,535047937	0,000144918	0,025567205
**Kif19a**	6,994888974	0,549714314	0,000166517	0,029068544
**Syndig1l**	6,65459875	0,517832345	0,000174901	0,029902589
**Casr**	4,988691236	0,711601482	0,000207125	0,03302854
**Cyb5d1**	7,030386252	0,483482965	0,00021898	0,034586384
**RP23-332E2.7**	3,729200235	-0,923109694	0,00022695	0,035506921
**Oprk1**	5,600679909	0,655892667	0,000239432	0,037109723
**Mir212**	3,357725456	-1,357881457	0,00025662	0,038892967
**Lox**	5,30762312	-0,534184372	0,000257973	0,038892967
**Tnni1**	3,584368874	0,923998415	0,000288401	0,041954719
**Rgs9**	8,038663943	0,44796957	0,000305048	0,043766787
**Gm4737**	3,95653166	-0,788699017	0,000316984	0,044843778
**RP24-390L20.5**	3,288289841	-0,897992141	0,000322475	0,044940499
**Gm10475**	4,581419909	-0,77032687	0,000330416	0,045663486
**Gm10143**	4,741120126	-0,632053476	0,000337194	0,046151657
**Ppp1r14a**	4,267036226	0,85165977	0,000351585	0,047403969
**Depdc1b**	3,045036993	1,258004209	0,000362975	0,048156581
**Bcas1**	10,9752945	0,446732673	0,000361205	0,048156581
**Gm9887**	3,284525342	-1,036015739	0,000379917	0,049574412
**Slc6a20b**	3,805006358	-0,896122701	0,000382629	0,049574412
**Ube4bos3**	4,06282201	-0,737740914	0,000377679	0,049574412

Supplementary Table 2: Primers used for RT-qPCR

Gene Name	Forward	Reverse
**Arc**	CTGAAGCAGCAGACCTGACA	CTCAGCAGCCTTGAGACCTG
**Plp1**	GCAAAGTCAGCCGCAAAACA	GCCCCTACCAGACATCTAGC
**Mag**	TTCTCAGGGGGAGACAACC	ACTCTCCTGGGGCTCTCAGT
**Mog**	CTGGCAGGACAGTTTCTTGA	AAAGAGGCCAATGGGAAATC
**Fos**	TACTACCATTCCCCAGCCGA	GCTGTCACCGTGGGGATAAA
**Fosb**	CTTCAACCAGCACAACCACC	TCTGCGAACCCTTCGCTTTT
**Gapdh**	CTTCTTGTGCAGTGCCAGC	GAGGTCAATGAAGGGGTCGT
